# The possible role of the endocrine disrupting chemicals on the premature and early menopause associated with the altered oxidative stress metabolism

**DOI:** 10.3389/fendo.2023.1081704

**Published:** 2023-02-14

**Authors:** Duygu Aydemir, Nuriye Nuray Ulusu

**Affiliations:** ^1^Koc University, School of Medicine, Department of Medical Biochemistry, Istanbul, Türkiye; ^2^Koç University Research Center for Translational Medicine (KUTTAM), Istanbul, Türkiye

**Keywords:** oxidative stress, premature menopause, endocrine disruptors, early menopause, antioxidant status

## Introduction

Endocrine-disrupting chemicals (EDCs) dysregulate hormone metabolism *via* interfering with estrogen, thyroid, and nuclear receptors such as peroxisome proliferator-activated receptors alpha and gamma (PPARα, PPARγ). EDCs can be either natural or manufactured chemicals such as aromatic hydrocarbons (PHAs), polychlorinated biphenyls (PCBs), phthalates, pesticides, flame retardants, phenols, and toxic metals exerting adverse health effects on both humans and wildlife (1). People are exposed to EDCs daily *via* inhalation, dermal contact, and digestion since these chemicals are found in almost all industrial products, including pharmaceuticals, cosmetics, toys, food packaging, medical devices, households, and plastics (2). EDCs are metabolized by liver, kidney, intestine, and skin esterases after exposure and secreted *via* urine and bile; however, some of these chemicals remain without metabolization and accumulate in the body (3, 4).

EDCs exert toxic effects even at low concentrations since they contribute to several pathogeneses, including infertility, endocrine dysfunction, impaired hormone metabolism, cancer, metabolic syndrome, obesity, diabetes, cardiovascular dysfunction, and reproductive and neurological disorders (5). On the other hand, the female reproductive system is directly affected by EDCs, for instance, premature aging of ovaries, and impaired follicle formation, growth, and activity. Moreover, endometriosis, premature births, polycystic ovary syndrome, infertility, epigenetic changes in DNA methylation, genotoxicity, and prolonged puberty are adverse outcomes of EDCs exposure in females (5–7). Dysregulation in the female reproductive system can lead to premature and early menopause associated with an increased risk of cardiovascular diseases, dementia, osteoporosis, mood disorders, sexual dysfunction, and mortality (8).

Since oxidative stress causes impaired folliculogenesis, meiosis, and ovulation, it can contribute to premature and early menopause. Furthermore, antioxidant administration improves the quality of maternally aged oocytes, blastocyst formation, and oocyte aging, preventing or delaying ovarian dysfunction (9). EDCs and their metabolized products contribute to oxidative stress by binding PPARs in the ovary and other tissues; also, adverse effects of the EDCs have been reported on the female reproductive system (10). Therefore, this paper discussed the possible role of EDCs-induced oxidative stress in premature and early menopause.

## The role of EDCs-induced oxidative stress in the female reproductive system

Oxidative stress is described by the imbalance between the antioxidant capacity and the production of reactive oxygen species (ROS) in the cell. Antioxidant defense in the cell is regulated *via* different antioxidant molecules and enzymes such as glutathione (GSH), glucose 6-phosphate dehydrogenase (G6PD), 6-phosphogluconate dehydrogenase (6-PGD), glutathione reductase (GR), glutathione s-transferase (GST), glutathione peroxidase (GPx), superoxide dismutase (SOD) and catalase (CAT). G6PD and 6-PGD take part in the pentose phosphate pathway (PPP), producing nicotinamide adenine dinucleotide phosphate (NADPH), which GR uses to convert oxidized glutathione (GSSG) to reduced glutathione (GSH). GSH/GSSG ratio is the primary biomarker for the oxidative stress, since increased levels of the GSSG indicate impaired redox balance in the cell. On the other hand, GPx and CAT detoxify hydrogen peroxide (H_2_O_2_), where SOD breaks down superoxide (O^2.-^) into water (H_2_O_2_) (11–13). Impaired oxidative stress status has been reported in the pathogenesis of various diseases such as cancer, diabetes, metabolic disorders, endocrine dysfunction, cardiovascular diseases, infertility, and neurological diseases, since ROS attack the DNA, lipid, proteins, and nucleic acids (14, 15). On the other hand, oxidative stress affects follicular fluid, oocyte maturation, ovarian steroid biosynthesis, ovulation, formation of blastocysts, implantation, embryogenesis, miscarriage, early birth, ovarian germ cell, and pre‐eclampsia, according to the literature (16).

Phthalates are a group of synthetic chemicals composed of alkyl diesters of phthalic acid found in almost all industrial products, such as cosmetics, toys, food wrappings, pharmaceuticals, households, and medical devices. Since phthalates are non-covalently bound to plastics, they can be easily released from the products to the environment (17, 18). Di(2-ethylhexyl) phthalate (DEHP), dibutyl phthalate (DBP), diethyl phthalate (DEP), and benzyl butyl phthalate

(BzBP) are metabolized into harmful byproducts such as monomethyl phthalate (MMP), monoethyl phthalate (MEP), monobutyl phthalate (MBP), mono(2-ethylhexyl) phthalate (MEHP), mono(2-ethyl-5-hydroxyhexyl) phthalate (MEHHP), mono(2-ethyl-5-oxohexyl) phthalate (MEOHP), monobenzyl phthalate (MBzP) and mono-n-octyl phthalate (MOP) in the body (19). DEHP and MEHP induce oxidative stress parameters, including 8-hydroxy-2′-deoxyguanosine (8-OHdG) and malondialdehyde (MDA) in the oocytes (20). Also, DEHP inhibits follicle growth and impairs endometrial cell function by inducing oxidative stress *via* decreased CAT, GPx (21) and SOD1 activity which is accepted as a biomarker for oocyte quality (22, 23). On the other hand, DEHP, MEHP, and DBP impair the cell cycle and induce apoptosis in the follicles by inducing oxidative stress, according to the literature (24).

Bisphenol A (BPA) is a plasticizer exerting endocrine-disrupting effects on the female reproductive system, such as reduced fertility, premature ovarian failure, inhibiting follicle growth, and decreased follicle counts (25). Moreover, 25 mg/kg/day BPA induced oxidative damage in the rat ovarian cells (26). BPA, bisphenol S (BPS), and bisphenol F (BPF) impaired the antioxidant status of bovine oocytes by reducing GPx and SOD activities (27). Pesticides are widely used chemicals in agriculture applications causing toxicity in the soil and water,

and other natural resources. Organophosphates and organochlorides cause the decreased estrous cycle, apoptosis in granulosa cells, clumping of oocytes, deletions in microvilli, inhibition of follicular growth, and damage in ovarian surface epithelium (OSE) *via* decreased GPx, SOD, CAT and GST activities in the rat and mice (28–30). Cadmium (Cd) is found in industrial products and agricultural activities, exerting endocrine-disrupting effects. Cd decreases antioxidant enzyme activities such as CAT and increases levels of MDA and H_2_O_2_ in the rat ovary. Also, Cd-induced oxidative stress cause reduced oocyte number and altered corpus luteum and oocyte tissue (31). On the other hand, persistent exposure to EDCs is associated with early menopause in women (32). Since different types of EDCs impair antioxidant capacity and increase oxidative stress in the female reproductive system, premature or early menopause can result from EDCs-induced oxidative stress.

## Discussion

Menopause is a natural gradual process that occurs in females between the ages of 45-55, resulting from the age-dependent decline in fertility. On the other hand, premature ovarian insufficiency (POI) or premature ovarian failure, also known as early and premature menopause, is characterized by ovarian failure before age 40, affecting 1% of women (33, 34). Decreased estrogen, increased follicle-stimulating hormone (FSH), and luteinizing hormone (LH) are characteristics of menopause. Compared to pre-menopausal women, increased oxidative stress and decreased antioxidant capacity have been reported in menopausal and post-menopausal women (35). For instance, reduced levels of SOD, CAT, GPx, GSH, vitamin C, and vitamin E have been reported in post-menopausal women compared to pre-menopausal females (36, 37). On the other hand, decreased estrogen levels during menopause contribute to impaired redox balance in females since estrogen has antioxidant effects (38).

Enzyme deficiencies, genetic disorders, environmental toxins, metabolic dysfunction, chemotherapy, radiotherapy, autoimmune diseases, and psychological factors contribute to the POI pathogenesis, which can be explained by the ovarian injury resulting from oxidative stress-induced apoptosis, inflammation, accelerated aging and mitochondrial dysfunction. For instance, increased MDA and decreased SOD and GPx activities have been reported in the POI (39, 40). Exposure to the EDCs including BPA, chromium, lead, cadmium, isoprene, methoxychlor (MTX), benzo(a)pyrene (BaP), 2-bromopropane, 2,5-hexanedione, ethylene glycol methyl ether (EGME), hexachlorobenzene, mancozeb, dicofol, carbosulfan, 4-vinylcyclohexene (VCH), butadiene methylcholanthrene (3MC), 2,2-bis(bromomethyl)-1,3-propanediol (BMPB), hexabromocyclododecane and dimethylbenzantracene (DMBA) is directly associated with the POI according to the literature (39).

Increased levels of urinary phthalates are tightly associated with the POI and decreased estradiol levels (41). BPA, DEHP, MHP, dichlorodiphenyltrichloroethane (DDT), MTX, 2,3,7,8-tetrachlorodibenzo-*p*-dioxin (TCDD), and bis-hydroxy methoxychlor (HPTE) exposure cause decreased estrogen and increased LH and testosterone contributing to the POI pathogenesis in the females (42). Three mechanisms, including follicle depletion, increased follicular recruitment, and impaired follicular maturation, are discussed as major contributors to EDCs-induced POI that all associated with the impaired oxidative stress metabolism (39). Enhanced oxidative stress or decreased antioxidant defense have adverse effects on both ovary and ovarian follicles. Dysfunction in the corpus luteum, altered follicular fluid, abnormal proliferation in the interstitial cells, apoptosis, decreased steroid synthesis in the granulosa cells, inhibition in the follicle growth and degeneration have been reported as oxidative stress-induced alteration in the ovary and ovarian follicles (43). On the other hand, antioxidant administration including melatonin, curcumin, resveratrol, quercetin, genistein, vitamin E, selenium, catalpol and hyperoside improved ovarian function and aging. For instance, SOD, CAT, GPx, GSH and thioredoxin reductase levels increased, whereas ROS, MDA, GSSG, 8-OHdG and H_2_O_2_ levels decreased upon antioxidant treatment (44).

## Conclusion

EDCs have been found in almost all types of industrial products such as cosmetics, toys, medical devices, food wrappings and household exerting adverse health effects by interfering with the hormone metabolism. Although EDCs are metabolized in the liver, kidney, skin and intestines, some parts of them remain without metabolization and accumulate in the body causing metabolic disorders, infertility, reproductive dysfunction, diabetes, cancer and neurological disorders. EDCs-induced oxidative stress leads to the ovarian aging, PCOS, apoptosis in follicles, reduced follicle reserve, impaired follicle formation, growth, and activity which are directly correlated with POI pathogenesis. POI affects %1 women under age of 40 associated with increased risk of mortality, cardiovascular diseases, metabolic disorders and diabetes. EDCs exposure have been associated with POI induced by different mechanisms including follicle depletion, increased follicular recruitment, and impaired follicular maturation correlated with impaired oxidative stress metabolism. On the other hand, antioxidant treatment improved oxidative stress-induced alteration in the ovary and ovarian follicles. In conclusion, EDCs-induced oxidative stress may result in the early and premature menopause which can be improved or eased *via* antioxidant treatment.

## Author contributions

DA and NU are responsible for the conceptualization and writing the manuscript. All authors contributed to the article and approved the submitted version.
